# Feature-by-feature comparison and holistic processing in unfamiliar face matching

**DOI:** 10.7717/peerj.4437

**Published:** 2018-02-26

**Authors:** Ahmed M. Megreya

**Affiliations:** Department of Psychological Sciences, College of Education, Qatar University, Doha, Qatar

**Keywords:** Holistic processing, Feature processing, Face matching

## Abstract

Identity comparisons of photographs of unfamiliar faces are prone to error but imperative for security settings, such as the verification of face identities at passport control. Therefore, finding techniques to improve face-matching accuracy is an important contemporary research topic. This study investigates whether matching accuracy can be enhanced by verbal instructions that address feature comparisons or holistic processing. Findings demonstrate that feature-by-feature comparison strategy had no effect on face matching. In contrast, verbal instructions focused on holistic processing made face matching faster, but they impaired accuracy. Given the recent evidence for the heredity of face perception and the previously reported small or no improvements of face-matching ability, it seems reasonable to suggest that improving unfamiliar face matching is *not* an easy task, but it is presumably worthwhile to explore new methods for improvement nonetheless.

## Introduction

Matching a face to a photo ID is a very common procedure in security settings although a large number of experimental studies showed that face matching is rather error-prone (e.g., for a review see Robertson, Middleton & Burton, 2015). For example, Bruce et al. and colleagues (1999) asked observers to match a target face presented above a target-present/target-absent 10-face line-up. All images were taken on the same day, under good lighting conditions, and were presented in a full-face view. In spite of these optimal conditions, which could be never met in any real-life scenario, participants’ performance was rather low, with an error rate of 30% for target-present and target-absent trials.

This low level of performance has been widely replicated (e.g., Bindemann et al., 2012; Megreya & Burton, 2008; Megreya, White & Burton, 2011; Megreya & Bindemann, 2013; Megreya & Bindemann, 2015; Megreya & Bindemann, 2017) even when the heavy demands of this 1-in-10 array methodology were remarkably reduced to a 1-in-1 face-matching task using a range of face-matching databases (e.g., Bruce et al., 2001; Burton, White & McNeill, 2010; Henderson, Bruce & Burton, 2001; Megreya et al., 2012; Megreya & Burton, 2008). In addition, high error rates were observed using a range of person-to-photo matching tasks, which resemble to a great extent the widely used verification procedures in security settings (Davis & Valentine, 2009; Kemp, Towell & Pike, 1997; Megreya & Burton, 2008; White et al., 2014). Furthermore, the performance of police and passport officers was rather low using the photo-to-photo and person-to-photo matching tasks (Robertson et al., 2016; White et al., 2014).

In a stark contrast, it has been known that the recognition of familiar faces is robust even under very challenging circumstances. For example, people can easily recognize familiar faces using highly degraded images (Burton et al., 1999; Watier & Collin, 2009), and even after very long retention intervals (Bahrick, Bahrick & Wittlinger, 1975). In addition, a short familiarization procedure was found to improve face matching performance using the 1-in-10 face matching task (Megreya & Burton, 2006; Megreya & Burton, 2007).

Accordingly, it has been proposed that unfamiliar face matching relies on image-based processes, while familiar face recognition engages a more sophistic and specialized type of processing (for a review, see Hancock, Bruce & Burton, 2000). For example, Megreya & Burton (2006) found no correlation between matching upright familiarized and unfamiliar faces; but there were strong positive correlations between matching upright and inverted unfamiliar faces and between matching upright unfamiliar faces and inverted familiar faces. As face inversion impairs configural, but not featural, processing (e.g., for a review see e.g., see Bartlett, Searcy & Abdi, 2003), Megreya & Burton (2006) suggested that unfamiliar faces in the matching tasks are treated as “images” or “simple visual patterns” and matched on this basis without domain-specific expertise. Consistently, Lobmaier & Mast (2007) found that the recognition of new faces, compared to old ones, relies relatively more on the processing of featural information, but with the course of familiarization, the significance of holistic processing increased.

The suggestion that unfamiliar face matching is a feature-based process, which relies on comparison of individual facial parts such as the eyes, nose or mouth, indicates that face matching could be improved by directing observers to the diagnostic features that are particularly beneficial for face matching. In fact, police and passport officers usually receive feature-by-feature comparison training (for reviews, see, e.g., Robertson, Middleton & Burton, 2015; Towler, Kemp & White, 2017), though the results of experiments that examined the effectiveness of this strategy are inconsistent (Berman & Cutler, 1998; Towler, White & Kemp, 2014; Towler, White & Kemp, 2017; Woodhead, Baddeley & Simmonds, 1979). Specifically, some experiments found that feature-by-feature comparison have no benefits for face recognition (Woodhead, Baddeley & Simmonds, 1979) and even impaired it (Berman & Cutler, 1998). Nevertheless, a more recent study by Towler, White & Kemp (2017) found that a feature similarity rating task could improve unfamiliar face matching. Specifically, a pair of faces was presented above a list of eleven facial features (ears, jawline, chin, check area, face shape, eyes, mouth area, forehead, mouth, nose, and scars/blemishes), and observers were asked to rate the similarity of those features. They were then asked to decide whether the face pair showed the same person or different people. The results demonstrated that this rating task improved matching accuracy on identity match, but not on non-match, trials. In addition, Towler, Kemp & White (2017) found no benefits for rating the similarity of personality traits that have been thought to rely on holistic processing (for a review, see, e.g., Coin & Tiberghien, 1997). Consistently, White et al. (2015) found that facial forensic examiners who received training on feature-by-feature analysis of facial images outperformed students in face-matching task. This superiority was especially strong when faces were turned upside down, suggesting that the feature-by-feature strategy employed by forensic examiners might be the locus of their expertise (White et al., 2015).

The same suggestion might also indicate that increasing the reliance of holistic processing during matching unfamiliar faces may improve participants’ performance. Holistic processing refers to the ability to recognize a face as a gestalt, rather than a collection of distinguishable features. This type of processing has been thought to underlie the robustness of familiar face recognition (e.g., for reviews see Maurer, Le Grand & Mondloch, 2002; Tanaka & Gordon, 2011). However, the results of previous experiments that examined the association between face recognition accuracy and holistic processing were mixed. For example, Wang et al. (2012) found that face recognition accuracy correlated with the extent to which observers processed the faces holistically (as indexed by the composite-face effect and the whole-part effect). Nevertheless, Konar, Bennett & Sekuler (2010) found a close to zero correlation between unfamiliar face matching accuracy and the magnitude of the composite face effect, which is a well-established measure of holistic processing.

The aim of this study was to examine the effectiveness of feature-by-feature comparison versus holistic processing on matching unfamiliar faces. Participants were instructed to use either a feature-by-feature matching strategy (where they could compare the features of two faces piece-by-piece) or a holistic matching strategy (where they could encode each face as a whole piece, rather than a collection of separable features) during matching pairs of unfamiliar faces. Because of the importance of face matching for security settings such as the country borders and the well-documented low levels performance on face matching, exploring new techniques for improving unfamiliar face matching is of particular importance and one of the central questions in the ongoing face identification literature (see, e.g., Robertson, Middleton & Burton, 2015).

## Method

### Participant

Forty under-graduate students from Qatar University volunteered to participate in this experiment (*M*_age_ = 20.5, *SD*_age_ = 0.7; 65% female). All participants had normal or corrected to normal vision. Ethical approval for participation in this study was provided by Qatar University’s institutional review board (QU-IRB 576-EA/16) and all methods were performed in accordance with the QU-IRB guidelines and regulations.

### Stimuli

A total of 120 Arab match/mismatch pairs of faces were used as stimuli in this experiment that were taken from an Egyptian face-matching database (Megreya & Burton, 2008). Each pair consisted of a target video still (taken by a video camera) and a test photograph (taken by a digital camera), which depicted the same face or two different persons. Pairing in mismatch pairs was made according to subjective overall similarity between the faces. All target and test images were taken at the same time and under the same lighting conditions. All faces were shown in a frontal view, with a neutral expression, and any extraneous background was removed. The size of each image was approximately 5 cm × 7 cm and all images were presented in grey-scale (for full details of these stimuli see (Megreya & Burton, 2008), where examples of face pairs can be found).

### Procedure

All participants were tested individually using an Apple laptop. Experimental software was used to present face-matching stimuli and to record participants’ responses. In two successive sessions, the participants’ task was to make match/mismatch decisions for the face pairs, which were presented randomly. Each pair was presented until a participant’s response was made by pressing two labelled keys in the standard computer keyboard. All participants were presented with 30 face pairs (15 matches and 15 mismatches) while half of the participants were given instructions that encouraged them to use either a feature-by-feature matching strategy or a holistic matching strategy. In the feature-by-feature matching instruction, participants were asked to match the faces by comparing the faces feature-by-feature. In the holistic matching instruction, participants were asked to look at the faces globally and not to focus on their parts. Immediately after these instructions, all participants were presented with 30 new face pairs (15 matches and 15 mismatches). Notably, match/mismatch pairs were counter-balanced across participants. Therefore, each face target was equally seen in match and mismatch pairs across the experiment, and there was no repetition of face identities across all trials. The face-matching tasks were also counter-balanced across the pre- and post-training sessions.

## Results

**Figure 1:**
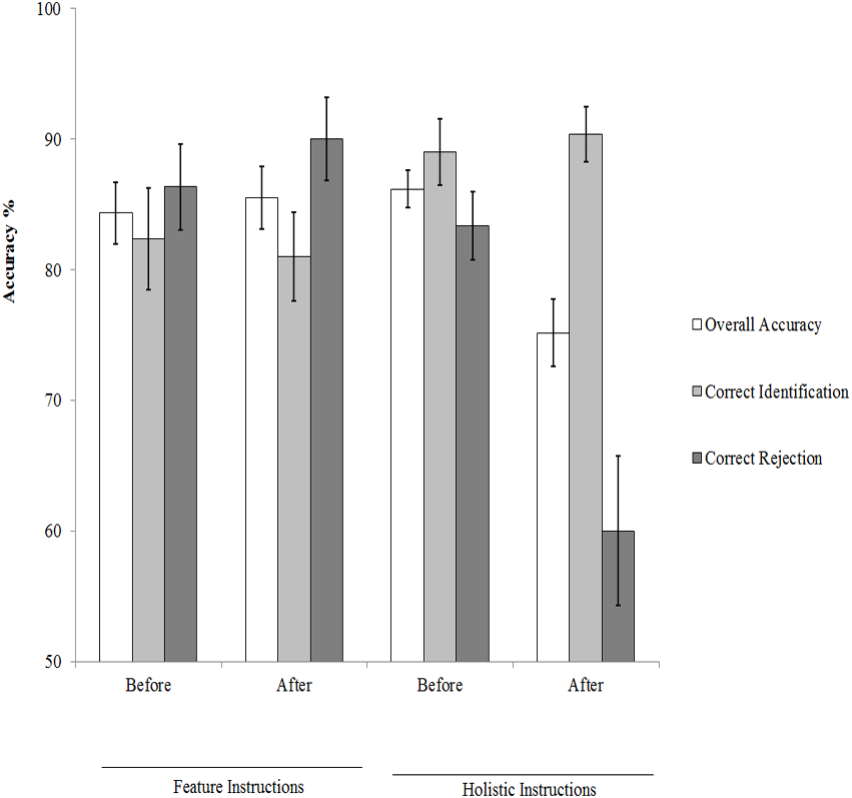
Participants’ performance in this experiment: accuracy measures.

The accuracy of face matching was calculated using three main measures. Correct Identification refers to the correct positive decision that the two faces are of the same person. Correct Rejection refers to the correct negative decision that the two faces depict two different people. Overall Accuracy refers to the total correct responses in match and mismatch pairs that were calculated by combining correct identification and correct rejections. Figure 1 shows the percentage averages for participants’ performances in this experiment. These data were subjected to three 2 (instructions: holistic processing versus feature-by-feature comparison) × 2 (training: before versus After) mixed-design, where the instructions variable was treated as a between-participant factor while training was treated as a within-participant factor. There were significant interactions between instructions and training for overall accuracy, *F*(1, 38) = 8.87, *p* < 0.01, ${\eta }_{p}^{2}=0.19$ and correct rejections, *F*(1, 38) = 14.50, *p* < 0.01, ${\eta }_{p}^{2}=0.27$, but not for correct identifications, *F* <1. A series of paired-sample *t*-tests between participants’ performances before and after training (with alpha adjusted to 0.05∕2 = 0.05 for two comparisons) revealed no significant effects for feature instructions on overall accuracy, *t* (19) = 0.40, *p* = 0.69, Cohen’ *d* = 0.09, and correct rejections, *t* (19) = 0.83, *p* = 0.42, Cohen’ *d* = 0.27. However, holistic instructions had detrimental effects on overall accuracy, *t*(19) = 3.80, *p* = 0.001, Cohen’ *d* = 1.17, and correct rejections, *t* (19) = 4.21, *p* < 0.001, Cohen’ *d* = 1.18.

**Figure 2:**
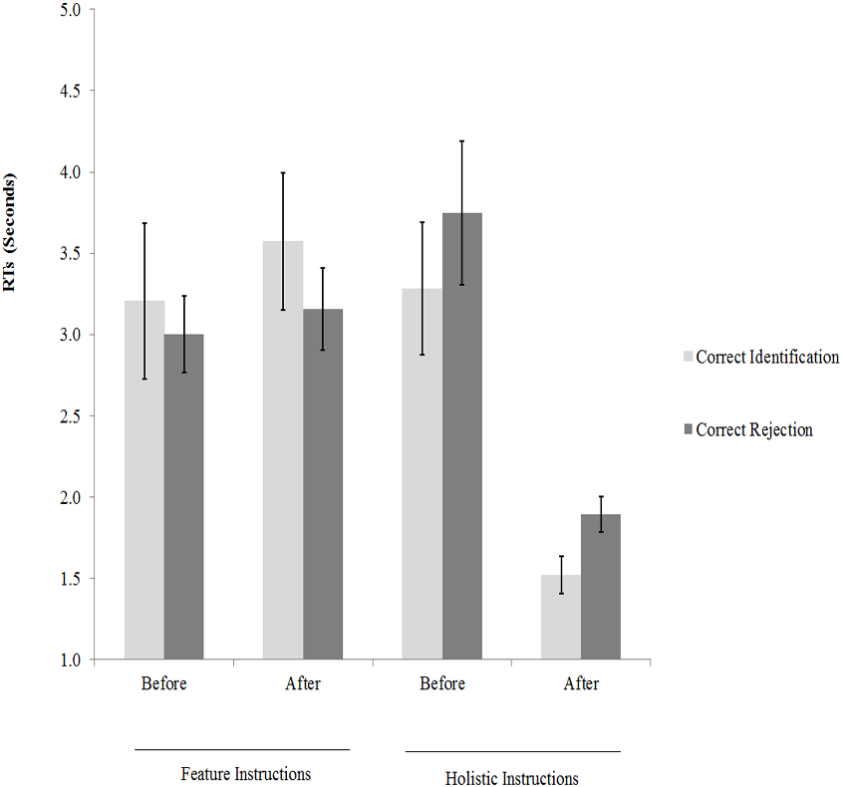
Participants’ performance in this experiment: response time measures.

In addition, the averages of medians of the response times were calculated for correct identification and correct rejection (see Fig. 2). These data were also subjected to two 2 × 2 mixed-design ANOVAs, which revealed interactions between instructions and training for correct identification, *F* (1,38) = 5.86, *p* = 0.02, ${\eta }_{p}^{2}=0.26$ and correct rejections *F* (1,38) = 10.69, *p* < 0.001, ${\eta }_{p}^{2}=0.28$. A series of paired-sample *t*-tests between participants’ performances before and after training (with alpha adjusted to 0.05∕2 = 0.05 for two comparisons) revealed no significant effects for feature instructions on correct identification, *t* (19) = 0.86, *p* = 0.40, Cohen’ *d* = 0.20, and correct rejections, *t* (19) = 0.49, *p* = 0.63, Cohen’ *d* = 0.09. However, participants were quicker to make the face-matching decisions after receiving holistic instructions using correct identification, *t* (19) = 4.63, *p* ≤ 0.001, Cohen’ *d* = 1.60, and correct rejections, *t* (19) = 4.44, *p* < 0.001, Cohen’ *d* = 1.24.

## Discussion

This experiment examined the effects of verbal instructions focused on feature-by-feature comparisons and holistic processing on unfamiliar face matching. The results showed that feature-by-feature comparison strategy did not benefit face-matching accuracy and latency (see Figs. 1 and 2). This finding converges with the results of an early study (Woodhead, Baddeley & Simmonds, 1979), in which a three-day training course emphasized on “isolated” facial features failed to make improvements on face recognition memory as well as face matching across different views and expressions. However, Towler, White & Kemp (2017) found that the similarity ratings of a list of specific facial features, prior to matching decisions, improved face-matching accuracy. Notably, there are several methodological differences that can explain the contrast between the results of the present experiment and those of Towler, White & Kemp (2017) study. For example, the present experiment used verbal instructions, whereas Towler, White & Kemp (2017) used a feature similarity rating task. Therefore, those verbal instructions might not cause participants to spend longer comparing individual features to the same extent as Towler, White & Kemp (2017).

The present experiment also found that verbal instructions focusing on holistic processing made participants’ performances faster (see Fig. 2), but they harmed accuracy (see Fig. 1). It has been commonly thought that faces are processed holistically rather than as a corpus of individual parts (e.g., for reviews see Maurer, Le Grand & Mondloch, 2002; Piepers & Robbins, 2012; Tanaka & Gordon, 2011; but for a different view see Burton et al., 2015). This holistic processing of faces is assumed to emerge very rapidly (Goffaux & Rossion, 2006; Richler et al., 2009). Consistently, encouraging observers to adopt a holistic processing strategy during matching faces significantly speeded performance on both match and mismatch trials. Importantly however, this response time advantage was associated with a lower level of face matching accuracy, especially in mismatch trials (see Fig. 1), suggesting that holistic processing strategy induced a speed-accuracy tradeoff.

## Conclusion

Previous studies reported that the face-matching performance of highly experienced passport and security officers was error-prone and similar to naïve participants (White et al., 2014; Wirth & Carbon, 2017). Existing data indicates that methods for improving matching accuracy in security settings are required (see, e.g., Robertson, Middleton & Burton, 2015; White et al., 2014). The current study indicates that verbal instructions focusing on feature-by-feature comparisons could not improve face matching. Conversely, verbal instructions focusing holistic processing could improve face matching latency but significantly harm accuracy. Given the genetic basis of face perception (Wilmer et al., 2010; Zhu et al., 2010) and the small or no improvements of face matching ability reported in this experiment and in previous studies (e.g., for a recent review see, Young & Burton, 2018), it seems reasonable to suggest that improving unfamiliar face matching is *not* an easy task, but it is presumably worthwhile to explore new methods for improvement nonetheless.

##  Supplemental Information

10.7717/peerj.4437/supp-1 Supplemental Information 1 Raw data

10.7717/peerj.4437/supp-2 Supplemental Information 2 Results of signal detection measures

## Additional Information and Declarations

### Data Availability

The following information was supplied regarding data availability: The raw data has been provided as a Supplemental File.
