# Supplementary material for: Feature-by-feature comparison and holistic processing in unfamiliar face matching
Source: PeerJ. 2018 Feb 26;6:e4437. doi: 10.7717/peerj.4437 (PMC5831152; doi:10.7717/peerj.4437)
Supplement: Supplemental Information 2 [file peerj-06-4437-s002.docx]

Signal detection measures

For completeness, the accuracy data was also transformed into signal detection measures of sensitivity (*d’*) and bias (*criterion*). The following table presents descriptive statistics these signal detection measures in this study. These data were subjected to two 2 x 2 mixed-design ANOVAs, which revealed significant interactions between instructions and training using *d*’ prime, *F* (1,38) = 9.09, *p* = 0.01, *η_p_^2^* = 0.19, and Criterion C, *F* (1,38) = 7.23, *p* = 0.01, *η_p_^2^* = 0.16. A series of paired-sample *t*-tests between participants’ performances before and after training (with alpha adjusted to 0.05/2 = 0.05 for two comparisons) revealed no significant effects for feature instructions on *d*’ prime, t (19) = 0.67, p = 0.51, Cohen’d = 0.20, and Criterion C, t (19) = 1.21, p = 0.24, Cohen’d = 0.36. However, holistic instructions tended to decrease sensitivity as indexed by d’ prime, t (19) = 4.05, p = 0.001, Cohen’d = 1.10, and increase biases as measured by Criterion C, t (19) = 2.73, p = 0.01, Cohen’d = 0.65.

|  | *d* prime | | Criterion c | |
| --- | --- | --- | --- | --- |
|  | M | SD | M | SD |
| *Feature Instruction* |  |  |  |  |
| Before | 2.51 | 1.03 | 0.10 | 0.52 |
| After | 2.63 | 0.96 | 0.29 | 0.52 |
| *Holistic Instruction* |  |  |  |  |
| Before | 2.61 | 0.71 | -0.20 | 0.56 |
| After | 1.80 | 0.74 | -0.57 | 0.58 |
